# Pangenome of *Serratia marcescens* strains from nosocomial and environmental origins reveals different populations and the links between them

**DOI:** 10.1038/s41598-018-37118-0

**Published:** 2019-01-10

**Authors:** Eduardo Abreo, Nora Altier

**Affiliations:** Laboratorio de Bioproducción, Plataforma de Bioinsumos, INIA Uruguay, Ruta 48 Km 10, Canelones, Uruguay

## Abstract

*Serratia marcescens* is a Gram-negative bacterial species that can be found in a wide range of environments like soil, water and plant surfaces, while it is also known as an opportunistic human pathogen in hospitals and as a plant growth promoting bacteria (PGPR) in crops. We have used a pangenome-based approach, based on publicly available genomes, to apply whole genome multilocus sequence type schemes to assess whether there is an association between source and genotype, aiming at differentiating between isolates from nosocomial sources and the environment, and between strains reported as PGPR from other environmental strains. Most genomes from a nosocomial setting and environmental origin could be assigned to the proposed nosocomial or environmental MLSTs, which is indicative of an association between source and genotype. The fact that a few genomes from a nosocomial source showed an environmental MLST suggests that a minority of nosocomial strains have recently derived from the environment. PGPR strains were assigned to different environmental types and clades but only one clade comprised strains accumulating a low number of known virulence and antibiotic resistance determinants and was exclusively from environmental sources. This clade is envisaged as a group of promissory MLSTs for selecting prospective PGPR strains.

## Introduction

*Serratia marcescens* is a Gram-negative rod-shaped bacterium of the *Enterobacteriaceae* family that has been isolated from different environmental and nosocomial sources. Its importance as an opportunistic human pathogen has been acknowledged in the last decades, when *S. marcescens* has been signaled as responsible for a range of symptoms in hospitalized patients including septicemia^1^, meningitis, infections of the urinary tract^2–4^, eyes^5^, bloodstream^6^ and organs of the respiratory apparatus^7^. Putative and confirmed virulence determinants have been recognized including the production of hemolysins (ShlA), proteases, siderophores and lipopolysaccharides^8^. Virulence factors of indirect action but whose presence increase virulence are related to bacteria motility and antibiotic resistance. However, some of these virulence factors, most notably those of secondary importance, may also confer competence for any strain to strive in different hosts like insects and plants surfaces, and also soil and water. A particular case that has drawn great interest is the presence of genetic determinants of intrinsic and acquired resistance to antibiotics, linked to the origin and spread of multidrug resistant strains (MDR) between the environment and hospitals, and the impact of this secondary factor as an enhancer of the virulence of the nosocomial strains^9–11^.

Being an ubiquitous microorganism, environmental S*. marcescens* has been isolated from water and soil, plants, insects, foods and machinery. Plant roots and their adjacent soil -the rhizosphere- can host *S. marcescens* and other species that positively interact with plants, enhancing nutrition, stress tolerance and health and therefore have been considered plant growth promoting rhizobacteria (PGPR)^12–14^. The rhizosphere of wild and cultivated plants is also known to host opportunistic human pathogens like *Burkholderia cepacia*, *Pseudomonas aeruginosa* and *Stenotrophomonas maltophilia*, although little is known about the virulence of these environmental strains relative to the clinical strains^15,16^. For *S. marcescens*, its double life-strategy as a human pathogen and as a PGPR has arisen doubts about its use in agriculture. In particular, there is a need to understand whether the nosocomial and environmental populations of *S. marcescens* can be clearly defined and whether strains promoting plant growth can be genotyped with high precision to undoubtly track them down in the field after their deployment in agricultural settings.

Whole genome sequencing (WGS) has emerged as an ultimate typing tool that fits any bacterial species, study type, and laboratory^17^. Whole genome multilocus sequence typing (wgMLST) has been proposed as a very useful and practical method based on WGS to distinguish strains within epidemic settings^18^ and between epidemic and unrelated specimens^17^. To apply MLST based on WGS, an allele database for the population of a bacterial organism must first be set.

In this work we have used the PGAdb-builder^19^, a recently available web-based tool, to create a pangenome allele database for this species and to apply wgMLST schemes to genomes of nosocomial, environmental and PGPR strains of *S. marcescens* available at GenBank to address questions regarding the link between origin, PGPR status and genotype. In addition, we explored the annotated genes in the genomes of selected *S. marcescens* to weigh the presence of some known virulence, antibiotic resistance and PGP determinants. The ultimate goal is to use the growing resource of genomic information to draw preliminary conclusions on the diversity and genetic make-up of *S. marcescens* populations and their suitability to be used as PGPR.

## Results

### Serratia marcescens pangenome

The average nucleotide identity (ANI) of whole genome sequences of 49 selected isolates, representing diverse environments and countries and including ATCC 13880 type strain, confirmed that 45 strains could be assigned to *S. marcescens* as they showed ANI values > 95.0% with *S. marcescens* type strain ATCC 13880 (Table 1). Within this group, most of the strains of nosocomial origin, all three strains from insects and 2 strains known as PGPR had ANI values between 95.0 and 96.0, while most environmental, 2 nosocomial and 2 PGPR strains had ANI values > 96.0 (Table 1). Among the four strains with ANI values below 95.0, that were not considered as *S. marcescens* and were no further analyzed, MSU97 -reported as a PGPR strain- had the lowest ANI value (93.77).

**Table 1:** Strains used in this study, source, country, GenBank genome accession, ANIm % and assembly parameters.

Strain Code^a^	Strain Name	Source of isolation	Country	Sequence Number	ANIm	Contigs N50-L50
**1**	2880STDY5683032	Nosocomial pathogen/blood	UK	GCF_001538705.1	95.64	251,419-7
**2**	2880STDY5682907	Nosocomial pathogen/blood	UK	GCF_001538645.1	95.67	278,529-7
**3**	2880STDY5683006	Nosocomial pathogen/blood	UK	GCF_001537785.1	95.67	340,665-6
**4**	SM1890	Nosocomial/urinary tract infection	Germany	GCA_900108825.1	95.34	94,867-24
*5*	LCT-SM213	Derived from CGMCC 1.1857	China	GCF_000264275.1	98.90	299,433-5
Na	W2.3	Sick fish	Malaysia	GCF_000292365.1	*94.44*	216,941-8
6	WW4	Paper machine	Taiwan	GCF_000336425.1	96.96	complete
7	VGH 107	Snake-bite wound	Taiwan	GCF_000342205.1	97.03	220,690--10
**8**	MC620	Stool of patient with bacteremia	USA	GCF_000418815.1	95.33	86,897-21
**9**	MC6001	Blood sample of individual with bacteremia	USA	GCF_000418835.1	95.32	85,735-22
10	EGD-HP20	Tannery waste water	India	GCF_000465615.2	97.00	189,845-10
11	Db-11	Derived from Db10/insect pathogen (Drosophila)	Sweden	GCF_000513215.1	96.07	complete
**12**	BIDMC 50	Bronchoalveolar lava	USA	GCF_000521905.1	95.45	685,952- 3
Na	H1q	Carnivorous plant phytotelma	Malaysia	GCF_000633335.1	94.44	345,525-4
Na	PH1a	Carnivorous plant phytotelma	Malaysia	GCF_000633555.1	94.44	374,527- 4
*13*	ATCC 14041	Unknown	USA	GCF_000695485.1	98.91	113,020- 15
14	ATCC 13880 ^T^	Pond water	Unknown	GCF_000735445.1	—	238,240- 7
**15**	FDAARGOS_65	Endotracheal aspirate	USA	GCF_000783915.2	95.64	complete
**16**	SM39	Nosocomial pathogen/septicemia	Japan	GCF_000828775.1	95.65	complete
***17***	90–166	Field-grown plant	USA	GCF_001007555.1	95.32	632,250- 4
**18**	CAV1492	Nosocomial pathogen/respiratory	USA	GCF_001022215.1	95.32	complete
***19***	RSC-14	*Solanum nigrum* plant	South Korea	GCF_001280365.1	95.47	complete
**20**	SmUNAM836	Bronchial sample/ obstructive pneumonia	Mexico	GCF_001294565.1	95.64	complete
21	B3R3	*Zea mays* plant	China	GCF_001417865.2	97.05	Complete
22	ano2	*Anopheles stephensi* gut contents	China	GCF_001853495.1	95.40	232,610- 4
23	Sicaria (Ss1)	*Apis mellifera* hemolymph	USA	GCF_001889685.1	95.42	105742- 18
Na	MSU97	Plant surface	Venezuela	GCF_001902635.1	*93.77*	197,047- 10
**24**	189	Feces of neonate/asymptomatic	Russia	GCF_001908015.1	95.64	100,707- 16
25	AS1	*Anopheles stephensi*	USA	GCF_001932655.1	95.40	3,366,121- 1
26	BJL200	Soil	Unknown	GCF_001940505.1	96.91	41,521- 45
27	19 F	*Atelopus zeteki* skin	USA	GCF_001975745.1	96.92	618,117- 3
28	1274	*Agave sisalana* plant	Brazil	GCF_002007925.1	95.40	1,503,251- 2
29	CAPREX_SY13	Ghanaian yam (plant)	England	GCF_002029205.1	98.94	498,095- 4
30	MEW06	Lake water	China	GCF_002094145.1	97.07	335,101- 6
**31**	D-1	Clinical/blood simple	USA	GCF_002104105.1	95.66	565,574- 3
32	Z6	Contaminated soil	China	GCF_002108655.1	97.07	652,363-4
33	S217	Petroleum contaminated soil	India	GCF_002205475.1	96.99	119,928- 14
**34**	UMH2	Bacteremia	USA	GCF_002220515.1	95.48	complete
**35**	UMH8	Bacteremia	USA	GCF_002220535.1	99.44	complete
**36**	UMH1	Bacteremia	USA	GCF_002220615.1	96.07	complete
**37**	at10508	Chronic obstructive pulmonary disease/lavage	Austria	GCF_002250685.1	95.33	120,371- 14
38	EGD-HP20_1	Tannery waste	India	GCF_002265665.1	96.90	25,890-65
***39***	KHCo-24B	*Gossypium hirsutum*	India	GCF_002592035.1	97.06	306,793-7
**40**	12TM	Pharyngeal secretions	Romania	GCF_002810285.1	95.66	complete
**41**	AR_0027	Clinical/patient associated	Unknown	GCF_002947235.1	95.67	complete
**42**	AR_0091	Clinical/patient associated	Unknown	GCF_002996885.1	95.62	complete
**43**	SMB2099	Clinical	Germany	GCF_900029885.1	95.65	complete
**44**	SM1978	Rectal swab at hospital admission	Germany	GCF_900108835.1	95.34	41,390-41
***45***	UENF-22GI	Cattle vermicompost	Brazil	SAMN03892645	98.92	*

^a^Origin of the strains is indicated  as nosocomial (**bold**), environmental (black), unknown (*italic*), PGPR reported status (***bold/italic***).

Na: strain code not assigned due to ANI < 95.

*UENF-22GI genome was downloaded from https://www.ncbi.nlm.nih.gov/Traces/wgs/?display = contigs&page = 1 and N50 – L50 parameters were not available.

The pangenome of *S*. *marcescens* built from 45 genome assemblies comprised a total of 19469 genes. The core genome -made of loci present in >95% of genomes- contained 3155 genes (16.2%), the dispensable genome contained 7847 genes (40.3%), while unique or exclusive genes -present in a single genome- were 8467 (43.5%). The automatically annotated genes were 4989, including core, dispensable and unique genes. The database identification number of this pangenome of *S. marcescens* is 1718436483 and can be accessed directly at http://wgmlstdb.imst.nsysu.edu.tw/disProfileDB.php?folder=1718436483.

### Classification of nosocomial, environmental and PGPR strains based on WGS MLST

**Figure 1:**
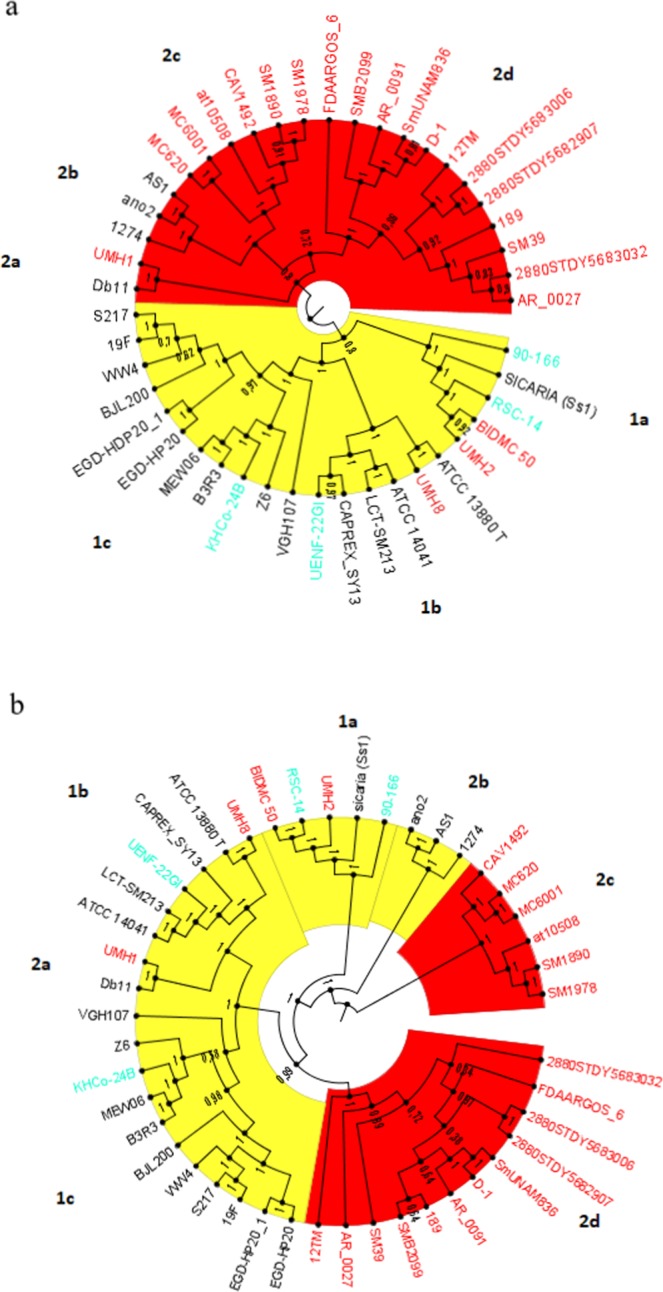
Dendrograms based on genetic distance between allelic sequences of **1a** core genes and **1b** whole genome genes of 45 *Serratia marcescens* strains representing nosocomial, environmental and PGPR strains. Taxa were colored according to the origin of the strain: clinical (red), environmental (black), PGPR (green) while clades were numbered and colored according to the assigned genomic type in the cgMLSTs: clinical (red), environmental/PGPR (yellow).

Two dendrograms with bootstrap values were generated based on allelic sequences of a maximum of 19469 genes by the Build_wgMLSTtree module, which uses PHYLIP program UPGMA clustering algorithm. The first dendrogram was based on genes that are included in the core genome (Fig. 1a). A central node divided the dendrogram in two sectors: one that gathered mostly strains of environmental origin (clade 1) and another that gathered mostly strains of nosocomial origin (clade 2). These two sectors were considered to represent environmental and nosocomial cgMLST respectively, and the subclades and strains in each sector were defined as environmental-type and nosocomial-type, independently from their origin.

Regarding the origin, nosocomial subclades 2c and 2d defined by cgMLST were integrated exclusively by strains from clinical origin. The sister nosocomial subclade 2b was integrated by three strains from environmental origin, including two strains from mosquitoes, and subclade 2a by one strain of nosocomial origin and one of environmental origin. Environmental clade 1 defined by cgMLST was formed mostly but not exclusively by strains of environmental origin, along with UMH8, UMH2 and BIDMC 50 of nosocomial origin. Also, environmental clade 1 could be separated in three sub-subclades (a, b, c). One of these subclades (1c) was formed exclusively by strains of environmental origin, while 1a and 1b contained 3 isolates of nosocomial origin. *S. marcescens* genomes with known PGPR activity were assigned to the environmental subclades 1a, 1b, 1c.

When the dendrogram was based on alleles of all genes (wgMLST), some re-ordering could be observed (Fig. 1b). Although in general subclades maintained their integrity, the most notorious rearrangement was the separation of nosocomial subclade 2b and 2c and environmental subclade 1a, which were detached from their original clades and formed independent clades. Besides, subclade 2a from the nosocomial clade, was relocated within environmental subclade 1b, adding one more strain of nosocomial origin (UMH1) to an environmental clade. The assignment of PGPR genomes to their subclusters was maintained for all strains.

### Analysis of genomes based on presence/absence of virulence genes

**Figure 2:**
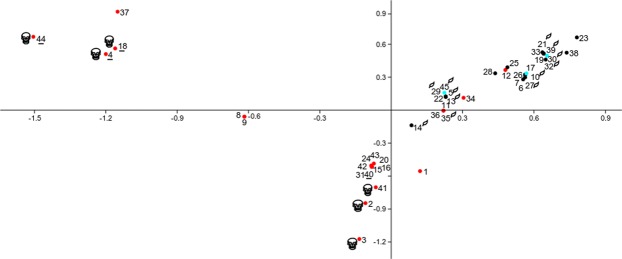
Correspondence analysis based on the presence/absence of 21 known virulence-related genes of *Serratia marcescens* strains of clinical (red) and environmental (black) origin, including strains reported as PGPR (green). Strains that accumulated higher numbers of known virulence, antibiotic resistance and PGP-related genes are indicated by skulls, underline and a leaf respectively.

An ordination of strains based on presence/absence of known genes related to virulence (Supplementary Table S1) was carried out by means of correspondence analysis (Fig. 2). This allowed for the clear separation of strains of clinical origin (red dots) in three groups, in the same fashion as these strains were grouped in the wgMLST tree: two main groups of strains of nosocomial origin separated along the horizontal axis whereas a group of 4 clinical isolates with an environmental wgMLST further separated along the vertical axis, forming a group with strains from environmental origin and those with reported PGPR status. Genomes showed different numbers of accumulated virulence, resistance and PGPR related genes (Supplementary Tables S1–S3; Fig. 2). The strains that ranked highest in accumulated number of virulence-related genes were in subclades 2c and 2d in Fig. 1a, those that ranked highest in antibiotic resistance genes were from clade 2c, while the strains accumulating more PGPR-related genes belonged with clade 1b and 1c. Genes coding for Beta-lactamases were present only in strains of nosocomial origin, mostly from clade 2c (Supplementary Table S2).

## Discussion

*Serratia marcescens* behaves as an opportunistic human pathogen in hospitals, where it affects immunocompromised patients^2–7,10^. At the same time, this species has been isolated from environmental sources like soils, water, insects and plants surfaces including the rhizosphere, where it can exert positive plant growth promotion effects^12–14^. The wide niche and functional diversity of this species raises questions about the genetic background that defines and separates nosocomial strains from environmental strains, and PGPR strains from other environmental strains. This is relevant to the discussions regarding the existence and distinction of nosocomial and non-nosocomial populations and the presence of genes related with virulence and antibiotic resistance in strains of this species that could be selected and used as PGPR.

In this work, we have attempted to assess differences between strains of *S. marcescens* of clinical and environmental origin based on the analysis of whole genome sequences of a panel of 45 strains - representing clinical and environmental populations from different countries- that were available at the GenBank assembly database.

This analysis implied a progressive approach in which strains were first grouped according to core genome MLST and whole genome MLST schemes. While the former was based on the allele variants of genes that are present in more that 95% of the tested strains, the latter was based on allele variants present also in the genes of the flexible genome or even exclusive genes, partly related to strain specific features like virulence and antibiotic resistance^20^.

A second step of the analysis was based on the presence/absence of some genes that are known to be involved in these traits. This added a bias towards clinical genomes regarding the presence of virulence/resistance determinants and towards PGPR genomes regarding the presence of PGP determinants. Therefore, although the correspondence analysis -based on the presence of virulence genes- was done with genomes of all origins, further comparisons and considerations were done within genomes of nosocomial and environmental origin by separate.

ANI value of 95.0 was used as a cut off threshold to circumscribe the species, which led to the exclusion of four strains that could hardly be considered part of *S. marcescens* species, including MSU97, a proposed PGPR strain of *S. marcescens*^21^. In a comparative genomic study of clinical and environmental *S. marcescens*^22^, this strain showed 1265 strain specific genes, a digital DNA to DNA hybridization (dDDH) value under 68%, and an isolated position in a phylogenetic tree based on 10 concatenated single copy core genes. In keeping with recent guidelines for bacterial species definition^23^, all this information supported our decision to not include MSU97 as part of the pangenome of *S. marcescens*.

The proposed pangenome of *S. marcescens* based on 45 strains from highly diverse nosocomial and environmental sources comprised 19469 genes, higher than 13614 genes reported from 205 clinical isolates in UK and Ireland^10^ and 16456 genes reported from a panel of 35 strains of *S. marcescens* from environmental and nosocomial origin, including MSU97^22^. Interestingly, only 16% of the genes were part of the core genome and 84% of the genes were either part of the flexible genome or were present in single strains, in nearly equal parts. This high number of exclusive and accessory genes can reflect the adaptability of *S. marcescens* to different niches and functions. Similar results regarding the high genomic variability of this species have been reported^10,20,22^.

The cgMLST scheme allowed for a segregation of most strains according to their origin, except in the case of 3 strains of nosocomial origin and 3 of environmental origin that were grouped with their alternative cgMLST. The position of the 3 strains that originated from hospitals within the environmental clades in the cgMLST and 4 strains in the wgMLST tree suggests that these nosocomial strains are derived from the non-clinical environment and therefore indicates that environmental strains of *S. marcescens* might develop as opportunistic pathogens in nosocomial infections.

Still, it is noticeable that the majority of strains from hospitals from different countries showed nosocomial MLSTs, which is indicative of the existence of a predominantly clinical population of *S. marcescens* that can be distinguished from the environmental counterparts. Within this population, the existence of two clades -2c and 2d- that are maintained in both MLST schemes implies that both subpopulations differ not only in the core genes but also in their particular accessory genes. This was also noted by Moradigaravard *et al*.^10^ in 2016, who found that nosocomial *S. marcescens* core genome revealed a highly structured population comprising distinct clades, with each clade showing a unique combination of accessory genes. In our study, although the analysis of virulence and resistance determinants was not exhaustive, nosocomial strains in clade 2c accumulated more antibiotic resistance genes and Beta-lactamase coding genes in particular. In this regard, multivariate analysis of strains based on presence/absence of virulence genes produced groups similar to those in the cgMLST and wgMLST: clade 2c of nosocomial strains, containing 6 virulent strains, was clearly separated from nosocomial strains that belonged to clade 2d and from the 4 strains of nosocomial origin but environmental wgMLST, that grouped again with the environmental strains. This last verification confirmed that although these 4 strains were obtained from diseased patients, not only their wgMLSTs but their virulence determinant, associated them with environmental strains. The significance of this finding is two folds: it confirms that at least some clinical strains of *S. marcescens* obtained from hospitalized patients are derived from the environment and therefore that some environmental strains might act as carriers of novel genes for the nosocomial population of *S. marcescens*, including unknown virulence and resistance determinants that were not considered in this study. Although it is known that the environmental resistome is distinct from the human -associated resistome, the environment can act as a reservoir of resistance determinants that can be present in human and clinical associated resistomes^24^ and opportunistic pathogens may represent a major conduit through which antibiotic resistance genes move between natural and nosocomial environments^25^.

On the other hand, the assignment of 3 strains from environmental sources to the nosocomial clade in the cgMLST tree suggests that the environment can host strains that are genetically more similar to nosocomial strains than to other environmental strains. However, when dispensable genes were added to produce the wgMLST tree, these three strains relocated from the nosocomial clade and formed an independent clade. This could reflect that these strains do not possess the set of dispensable genes that characterize the other strains from nosocomial origin and MLST. Two of these three strains (ano2 and AS1) were isolated from *Anopheles stephensi* – the malaria vector- and are known to be commensal strains capable of exerting control not on the vector but on *Plasmodium*, the causal agent of malaria^26,27^. Strain ano2 is known to have many predicted virulence factors including hemolysins, chitinases, flagella formation and lipooligosacharide^27^, but lacks genes encoding prodigiosins, which can be lethal to mosquitos. These highly specific symbionts might then conserve some of the pathogenicity related genes, needed to cope with the mosquito blood meals and to outcompete other species in the mosquito gut microbiota, as part of their core genome, but lack other genes that might damage the host, which would be no longer present in the dispensable genome. Such specific combination of attributes might explain why these strains separated from the nosocomial clade when all genes were considered.

According to Bruto *et al*.^28^, the PGPR status is dependent on the accumulation of a variable assortment of genes. It is noteworthy that the strains that accumulated more PGP-related genes belonged to subclade 1c and 1b in the MLST trees. Subclade 1c contains only environmental strains and one reported PGPR strain. Taken together, this could be an indication that clade 1c is a prospective PGPR clade, with sequence types less probable to be found in nosocomial environments. However, the inclusion of more genomes from clinical origin is necessary to challenge this hypothesis. The fact that no other PGPR have been found within this clade might be a consequence of these strains not yet being tested for their PGP activity. Therefore, clade 1c, with its particular accumulation and assortment of PGP genes and the low number of known virulence and resistance determinants is thought to represent a promissory safe MLST for finding more strains with PGP traits. Yet, because unknown virulence and resistance genes may be present but unnoticed in any environmental strain^24^, it is foreseen that more detailed bioinformatic studies based on WGS should be implemented on promissory strains from this subclade, to weigh this possibility^29^.

In conclusion, the proposed pangenome of *S. marcescens* and both cgMLST and wgMLST schemes can be used to type strains of this species and assign them to environmental or nosocomial MLSTs and clades, with wgMLST scheme producing a more strict segregation, probably reflecting specific functions that reside in genes of the dispensable genome.

The fact that some strains from nosocomial origin had environmental MLSTs highlights the potential role of opportunistic pathogens -like *S. marcescens-* in the gene flow between the environment and hospitals.

One subclade named 1c was found to comprise only environmental strains, including one reported as PGPR. Some strains in this subclade were also the ones accumulating more PGP traits and fewer known virulence and antibiotic resistance determinants and could therefore be signaled as a promissory clade from which obtain more PGPR. Because strains in this clade may possess unknown virulence or antibiotic resistance genes that have not yet been annotated, their presence should be addressed by different approaches to further sustain the use of strains from this subclade as PGPR.

## Methods

### Genomes

Genomes of *S. marcescens* assembled as contigs, scaffolds or complete genomes were downloaded from the National Center for Biotechnology Information (NCBI) Assembly database (https://www.ncbi.nlm.nih.gov/assembly). A total of 49 assemblies comprising 23 nosocomial strains, 24 environmental strains and 2 of unknown origin including reference strains ATCC 13880^T^ were downloaded (Table 1). Nosocomial strains were chosen randomly from a range of countries, with some specific strains included for their known virulence or prevalence based on bibliography^9,10^. Environmental strains were chosen also from a range of countries and sources (lakes, plants, insects, soils) with 5 strains included for their reported PGPR status (Table 1).

Average nucleotide identity was calculated by MUMer algorithm (ANIm) for 48 strains in relation with ATCC 13880 type strain with JSpecies software^30^ to verify that they could be considered *S. marcescens* (ANIMm > 95). Only strains with ANIm higher than 95 % were assigned a strain code (Table 1) and used in subsequent analyses.

### Building a *S. marcescens* pangenome

To build a pangenome for *S. marcescens*, 45 assemblies with ANI > 95.0 were first uploaded to the Build_PGAdb module of the PGAdb-builder server^19^ freely available at http://wgmlstdb.imst.nsysu.edu.tw. In this module, genes were annotated using Prokka and paralogous genes were excluded from the pan-genome allele dataset by default. Each orthologous cluster consisted of a protein family (a gene) with 95% sequence identity (default parameter). For allele calling, sequences in a locus with one or more mismatched nucleotides between each other were defined as different alleles. A pie chart was produced showing the number of genes in the core genome (genes shared by at least 95% of the genomes), number of genes exclusive or unique genes (present in a single genome) and the number of genes present in more than 1 but less than 95% of the genomes (the flexible genome). Besides, a list of annotated genes and the sequences of each allele of each annotated gene were produced.

### Building MLST trees based on whole genome sequences

Two MLST trees based on WGS were built for the same pangenome: one scheme included genes from the core genome (cgMLST) and the other scheme included all genes in the pangenome (wgMLST). For this, the same 45 genomes were uploaded on the module Build_wgMLSTtreeof the PGAdb-builder server. The uploaded genomes were compared with the built allele database (PGAdb) using BLASTN23 with parameters set by default. Because the same set of assemblies was used to create the pangenome and to build the wgMLST trees, all the alleles of any given gene were expected to be present in at least one sequence. After the allele finding process was finished for each scheme, the allelic sequences in each scheme were created. A dendrogram with bootstrap values, which is calculated by the ETE tool kit24, was then constructed from allelic sequences with the PHYLIP program through use of UPGMA clustering algorithm by default. For each scheme, the Build_wgMLSTtree produced files for presence/absence of loci, alleles present in each loci of each strain, a distance matrix and a dendrogram based on the genetic relatedness of the genomes. Dendrograms were further edited with FigTree^31^.

### Multivariate analysis of genomes based on presence/absence of genes

Genes that are known to be linked to virulence, plant growth promotion and antibiotic resistance were selected from bibliography^11,22,28^ and used to search in the Prokka annotated gene list produced by the PGAdb_builder. An ordering of strains based on the presence/absence matrix of virulence genes was achieved by multivariate analysis with software PAST^32^. The strains accumulating the highest number of virulence, resistance and plant growth promotion genes were highlighted on the graph.

## Supplementary information

S1

S2

S3

## Data Availability

The datasets generated and analyzed during the current study are available in the following links: Pangenome: http://wgmlstdb.imst.nsysu.edu.tw/disProfileDB.php?folder=1718436483 cgMLST_tree, presence/absence of genes and allele database: http://wgmlstdb.imst.nsysu.edu.tw/diswgProfiling.php?folder=12569572 wgMLST_tree, presence/absence of genes and allele database: http://wgmlstdb.imst.nsysu.edu.tw/diswgProfiling.php?folder=1699143652.
